# Diet and prey selection by snow leopards in the Nepalese Himalayas

**DOI:** 10.1371/journal.pone.0206310

**Published:** 2018-12-05

**Authors:** Bikram Shrestha, Joxerra Aihartza, Pavel Kindlmann

**Affiliations:** 1 Department of Biodiversity Research, Global Change Research Institute CAS, Brno, Czech Republic; 2 Institute for Environmental Studies, Faculty of Science, Charles University, Prague 2, Czech Republic; 3 Biodiversity and Environment Enhancement Nepal, Kathmandu, Nepal; 4 Department of Zoology and Animal Cell Biology, Faculty of Science and Technology, University of The Basque Country, UPV/EHU, Leioa, The Basque Country, Spain; Université de Sherbrooke, CANADA

## Abstract

Visual attractiveness and rarity often results in large carnivores being adopted as flagship species for stimulating conservation awareness. Their hunting behaviour and prey selection can affect the population dynamics of their prey, which in turn affects the population dynamics of these large carnivores. Therefore, our understanding of their trophic ecology and foraging strategies is important for predicting their population dynamics and consequently for developing effective conservation programs. Here we concentrate on an endangered species of carnivores, the snow leopard, in the Himalayas. Most previous studies on snow leopard diet lack information on prey availability and/or did not genetically check, whether the identification of snow leopard scats is correct, as their scats are similar to those of other carnivores. We studied the prey of snow leopard in three Himalayan regions in Nepal (Sagarmatha National Park (SNP), Lower Mustang (LM) and Upper Manang (UM) in the Annapurna Conservation Area, during winter and summer in 2014–2016. We collected 268 scats along 139.3 km linear transects, of which 122 were genetically confirmed to belong to snow leopard. Their diet was identified by comparing hairs in scats with our reference collection of the hairs of potential prey. We determined prey availability using 32–48 camera-traps and 4,567 trap nights. In the SNP, the most frequent prey in snow leopard faeces was the Himalayan tahr in both winter and summer. In LM and UM, its main prey was blue sheep in winter, but yak and goat in summer. In terms of relative biomass consumed, yak was the main prey everywhere in both seasons. Snow leopard preferred large prey and avoided small prey in summer but not in winter, with regional differences. It preferred domestic to wild prey only in winter, and in SNP. Unlike most other studies carried out in the same area, our study uses genetic methods for identifying the source of the scat. Studies solely based on visual identification of samples may be strongly biased. Diet studies based on frequency of occurrence of prey tend to overestimate the importance of small prey, which may be consumed more often, but contribute less energy than large prey. However, even assessments based on prey biomass are unlikely to be accurate as we do not know whether the actual size of the prey consumed corresponds to the average size used to calculate the biomass eaten. For example, large adults may be too difficult to catch and therefore mostly young animals are consumed, whose weight is much lower. We show that snow leopard consumes a diverse range of prey, which varies both regionally and seasonally. We conclude that in order to conserve snow leopards it is also necessary to conserve its main wild species of prey, which will reduce the incidence of losses of livestock.

## Introduction

Large carnivores (lion, tiger, common and snow leopard etc.) are often at risk of extinction. This, together with their visual attractiveness for people, makes them flagship species in conservation biology. Their hunting behaviour and patterns in their selection of prey may profoundly affect the population dynamics of their prey [1–3], which in turn affects the population dynamics of these large carnivores. In addition, if large carnivores attack domestic animals, the local people turn against them and retaliate, which causes a mixture of both positive and negative indirect interactions between wild prey on one side and cattle and other domestic animals on the other side, all sharing a common predator [4–6]. Therefore, an understanding of the trophic ecology and foraging strategies of large carnivores is important for predicting their population dynamics and developing effective conservation programmes.

Here we concentrate on an endangered species of carnivores, the snow leopard (*Panthera uncia*), which lives in alpine and sub-alpine meadows at altitudes of 2,500–5,500 m above sea level. It favours a steep terrain, well broken by cliffs, ridges, gullies and rocky outcrops [7, 8]. Being included in Appendix I of CITES, snow leopard is categorized as Vulnerable in the IUCN Red List, with a global population of 4,000–7,800 mature individuals [9]. Its survival is currently threatened by human activities, including habitat loss, retaliatory killings for livestock predation and poaching, primarily associated with the trade in pelts, bones and other body parts used in oriental medicine [9, 10].

A single snow leopard requires about 1.5 kg of meat per day, which is equivalent to 20–30 adult blue sheep per year [7, 11, 12]. Snow leopard diet is determined by analysing the remnants of prey in their scats [13–19]. These analyses indicate that snow leopards mainly eat ungulates (*Ovis* spp. and *Capra* spp.), but will also eat smaller prey such as marmot (*Marmota* spp.), hare (*Lepus* spp.) and/or pika (*Ochotona* spp.) [13–16, 20]. According to these analyses, wild ungulates constitute the main part of the diet of snow leopard (25–90%), followed by livestock (0–67%) and smaller prey such as rodents and birds (1–40%) [17, 21–23]. However, most of the data used in these analyses are not reliable for two reasons, as is explained below.

The first reason is that scats of sympatric carnivores, like wolf, common leopard, red fox, golden jackal etc., may be very similar and therefore prone to misidentification [21, 24, 25]. Actually, DNA analyses of snow leopard scats confirms the visual identification in only 40–60% of the cases [21, 23, 26], which suggests that the results of diet analyses obtained using conventional methods may be strongly biased. When the scats are not genetically identified the results are biased towards other species such as marmots (*Marmota* spp.) or bharal (*Pseudois nayaur*) [20, 27], but when identified using genetic markers, a much larger percentage of large–bodied ungulate prey is recorded [21, 23, 26].

The second reason is that of the studies on snow leopard foraging behaviour, only those of Lyngdoh et al. [20] and Chetri et al. [28] compare snow leopard diet with prey availability, and many other studies either lack information on prey abundance [13, 14, 19, 21] or provide data for only the largest prey available or data from a single season [15, 16, 18, 22, 23]. However, as the gut contents reflects both availability and the predator’s preference for different species of prey, these two factors must be strictly distinguished [29].

Here we take into account both of the above mentioned reasons for possible bias. We study the selection of prey by snow leopards based on their scats by consistently using genetic methods to determine the scat depositor and always determining the abundances of all potential species of prey in the territory of the scat depositor. We also distinguish winter and summer seasons, as prey preference may reflect seasonal effects. We chose three regions in the Nepalese Himalayas, one of the most important areas of snow leopard occurrence. We ask, whether snow leopards:

1. consume prey in direct proportion to its availability,
2. show any selection regarding prey size,
3. distinguish between wild and domestic prey,
4. show seasonal or regional differences in their selection of prey.

## Material and methods

### Study area

The study areas were located in three key snow leopard areas in Nepal: (i) Lower Mustang (LM), (ii) Upper Manang (UM)–both in the Annapurna Conservation Area (ACA), and (iii) Sagarmatha National Park (SNP).

ACA (N 28°47′ to N 28.78° and E 83°58′ to E 83°58′, ca 7629 km^2^) is located in west-central Nepal, in a transition zone between the moist southern Himalayan slopes and the high deserts in Tibet. This area borders the dry alpine deserts of Dolpo and Tibet in the north, the Dhaulagiri Himal in the West, the Marshyangdi Valley in the East and valleys and foothills surrounding Pokhara in the South. ACA contains some of the world's highest peaks, over 8,000 m, and the world's deepest valley, that of the Kali Gandaki River [30, 31]. LM and UM areas are mostly covered by alpine grassland (4,500–5,000 m) and subalpine scrubland (4,000–4,500 m). *Juniperus squamata* dominates the scrub community on gentler slopes, while rocky areas and steeper slopes are dominated by *Caragana gerardiana*, *C*. *brevispina*, *Rosa sericea*, *Ephedra sp*. and *Lonicera sp*. Above 4,800 m, the vegetation is meagre and dominated by *Rhododendron anthopogon*, *Potentilla biflora* and *Saxifraga sp*. Patches of *Pinus excelsa–Betula utilis–Juniperus indica* forests occur along the riverbanks.

The core areas of snow leopard in the ACA include Jomsom, Muktinath and Jhong in LM (ca 100 km^2^) and Proper Manang, Khansar and Tanki Manang in UM (ca. 105 km^2^). By using camera traps and faecal DNA tests, we identified five other large mammalian predators: common leopard (*Panthera pardus*), Tibetan wolf (*Canis lupus*), lynx (*Lynx lynx*), golden jackal (*Canis aureus*) and red fox (*Vulpes vulpes*). Blue sheep (*Pseudois nayaur*) and musk deer (*Moschus chrysogaster*) were the only wild ungulates in this area. Among small mammals, we recorded Royle's pika (*Ochotona roylei*), stone marten (*Martes foina*) and Sikkim vole (*Alticola sikkimensis*), all of which are potential prey of snow leopard. In 2011, there were 2251 inhabitants and 713 households at the three study sites in LM [32], owning 7,580 domestic animals, including yak, cattle, horses and goats [33]. In Manang, the human population is lower and declining, with 299 households and 1,264 inhabitants registered at the three study sites in 2011 [34]. Accordingly, livestock pressure is also lower there, with 3,975 yak, cattle, horses and goats registered in 2016. Both LM and UM are primary tourist and trekking destinations, receiving 18,000–30,000 and 14,000–22,000 visitors per year, respectively. In both areas, unregulated collection of timber, fodder, fuel wood, leaf litter and medicinal and/or aromatic plants, in synergy with livestock grazing and roads have negatively affected the biodiversity of the forest and rangeland. Natural disasters, such as forest fires and avalanches have also negatively affected the wildlife. Heavy snowfall and avalanches in 2014 and 2015 killed ~ 200 yaks and ~ 50 blue sheep in these regions.

SNP (N 27°45′ to N 28°07′ and E 86°28′ to E87°07′, area 1148 km^2^) is in the Solo Khumbu district in north-eastern Nepal. It includes the upper catchment areas of the Dudh Koshi and Bhote Koshi rivers and the Namche, Khumjung and Chaurikharka Village Development Committees (VDCs). Below the snow line, at 3,000 m, we recorded alpine plants, shrubs and coniferous vegetation, such as pine *Pinus* spp., fir (*Abies spectabilis*), juniper (*Juniperus* spp.), birch (*Betula utilis*) and rhododendron (*Rhododendron* spp.). In SNP, we recorded snow leopard, common leopard, musk deer and Himalayan tahr (*Hemitragus jemlahicus*). For our study in SNP, we chose four major valleys along the rivers Gokyo, Namche, Phortse and Thame, covering ca. 100 km^2^. These four areas were inhabited by 1,031 households and 3,452 inhabitants in 2011 (2.87% annual growth rate; [32], and by 5,332 domestic animals, including yak, cattle and horses in 2015. Every year, 30,000 to 37,000 tourists and trekkers visit the SNP [34]. This region is not yet connected with the rest of the world by road, but there are three helipads there: at Namche, Khumjun and Phortse. Other negative effects on wildlife and their habitats include collection of fodder, fuel wood, leaf litter, mushrooms and medicinal and aromatic plants, as well as livestock grazing, stone-mining and livestock dung collection from shrub and grassland areas.

### Scat sampling

From 2014 to 2016, we established transects covering a total linear distance of 139.3 km (102 transects, mean length 776 m, range 400–1,200 m, SE = 34.5) in both the summer and winter seasons at the three study sites. These transects were of the type used by the Snow Leopard Information Management System (SLIMS; [35]). With the aid of 1:50,000 topographic maps, we established transects along land forms such as ridgelines, narrow valleys, trails and cliff-edges, where snow leopards are likely to walk and leave signs [36–38].

Along these transects, we collected 261 putative snow leopard scats. For each scat found, a small portion was placed in a 15 ml plastic tube with silica desiccant to be used in the DNA analysis [39] and the remaining part of the scat was sun-dried, labelled and stored in polythene bags. These samples were genotyped to identify the species following Janecka et al., 2008. Scat DNA was extracted using Qiagen QIAamp DNA Mini Stool Kit [40], and a ~148 bp segment of mitochondrial cytochrome-b was PCR-amplified using the carnivore-specific primers CYTB-SCT-PUN-R’ (5’-AGCCATGACTGCGAGCAATA) and CYTB-SCT-PUN-F’ (5’-TGGCTGAATTATCCGATACC) [41].

The PCR products were run on a 2% agarose gel, stained with ethidium bromide, and visualized under ultraviolet light. The PCR of each scat sample was performed twice and any result that was based on a single positive was repeated for a third time. Only duplicate positives were considered as positive, whilst duplicate negatives were considered negative for snow leopard. For identification of general carnivores, a ~150 bp segment of mitochondrial cytochrome-b was PCR-amplified using the carnivore-specific primers set (CYTB-SCT-F: 5' AAACTGCAGCCCCTCAGAATGATATTTGTCCTCA 3' and CYTB-SCT-R: 5' TATTCTTTATCTGCCTATACATRCACG 3') [41]. The PCR products were run on a 2% agarose gel, stained with ethidium bromide, and visualized under ultraviolet light. Carnivore ID positives were sequenced for identification of carnivore species using the BLAST method.

### Diet analysis

**Fig 1:**
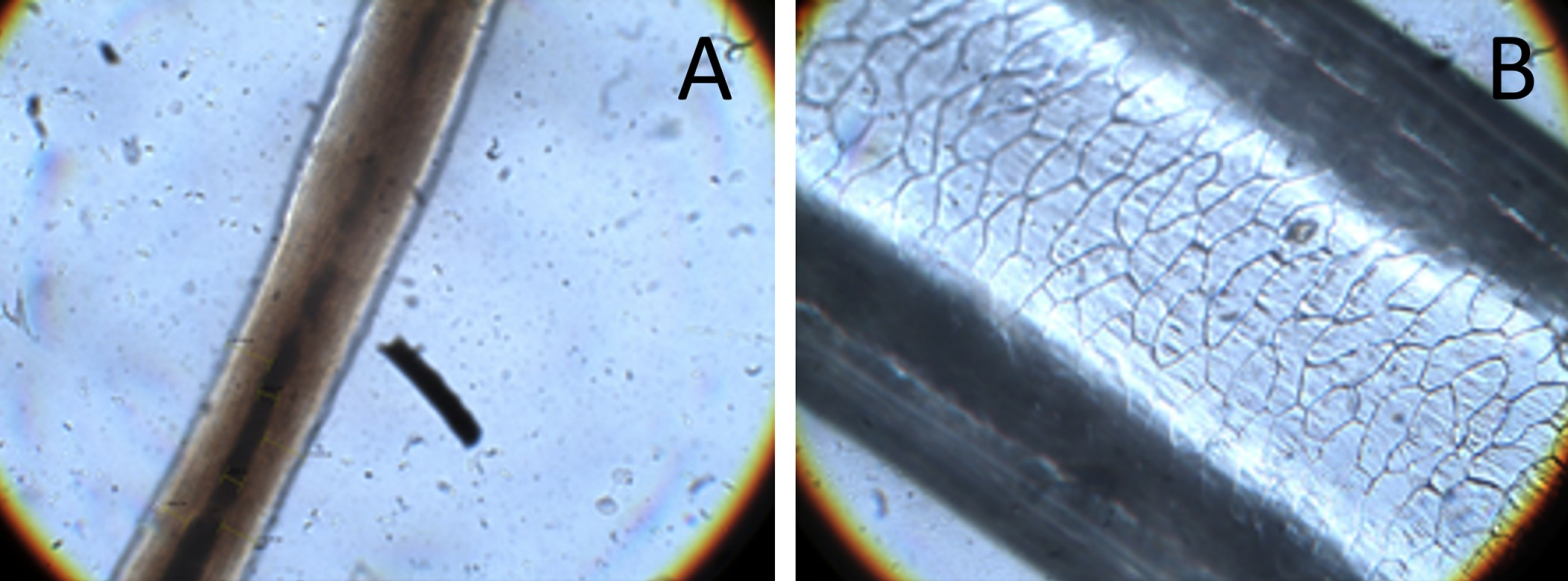
Microscopically prepared hair: A—Medulla of Yak (X40); B—Medulla of Blue sheep (X 40).

Using the remains of 11 potential species of wild prey found in the field and those of 6 domestic species of mammal gathered from the villagers, we built a collection of hair samples and an identification key, following the method described in [13], [42] and [43]. This key is based on microscopic examination of cuticle scales and medullary types, thickness of cortex and medulla, and medullary index, among other characters. The diet of snow leopard was then determined from the collected scat samples by comparing the hairs found in the scats with our reference collection of hair samples (Fig 1).

Each scat was soaked overnight in liquid Dettol mixed with water and then washed carefully over a sieve with a 1 mm mesh. Remains like bones, teeth, hooves, hair and feathers were removed, air-dried and stored. To avoid biases, we randomly selected twenty hairs from each scat [44] and mounted them on microscope slides, prior to identifying the prey using the identification key and reference collection.

We calculated the following values: relative frequency of occurrence (RF) (no of occurrences of each food type (species) when present/total no. occurrences of all food items ×100) [45] and relative biomass consumed (RBC) following [46]. Relative biomass consumed (kg): $RBC(S_{i})=100*({RF(S}_{i})*Y_{i})/\sum_{j=1}^{n}({RF(S}_{j})*Y_{j}),$ where *Y*_*i*_ comes from the correction equation *Y*_*i*_ = 1.980 + 0.035 *X*_*i*_ developed in [46], and *X*_*i*_ is the average body weight of the prey species obtained from [46].

### Prey availability

The relative abundances of potential prey in the study areas were obtained using camera-trap surveys. For this, we used remotely triggered camera-traps (Bushnell HD camera, passive infrared detector Trophy Camera) placed along well-defined, narrow ridgelines or valleys, or immediately adjacent to frequently scent-sprayed rocks and scrapes. Study areas were divided into 4×4 km grids, which correspond to the average home range size of female snow leopards [38, 47]. One or two camera stations were set up in each grid cell, with one or two camera-traps per station, deployed at 2–3 m from the anticipated path of animals. These camera-traps were checked and batteries changed approximately every 7 to 10 days. The monitoring with 32–48 camera-traps resulted in 4,567 trap nights during two trapping sessions at each site, including two winter (October-January) and two summer seasons (April-August).

Prey availability was assessed based on the number of independent sightings/captures by camera traps for each species per unit of sampling effort [48–50]. Photographs taken at instants differing by more than one hour were considered as independent sightings. If the instants differed by less than one hour, we compared colour patterns or other distinctive marks of the animals to determine, whether they were different or not.

In the case of prey, we have used a different approach. Whenever a single individual or a group of ungulates appeared, we considered it as a single “capture event” and we used the numbers of these “capture events” per 100 trap nights in subsequent analyses. This is better than attempting to count the number of individuals in the herd, as estimating this from camera traps may be biased or even impossible, because the camera will only rarely (if at all) capture the whole herd. The relative availability of potential prey was then assessed using the Relative Abundance Index (RAI) expressed as the percentage of capture events of species *I* relative to the total of capture events of all species. This index has previously proven reliable for assessing prey availability for tigers in Asia [48] and leopards in Africa [51].

### Prey selection

We studied prey selection by comparing a sample of consumed prey units with a sample of available prey, both recorded at population level (design I-A, following [52]). Independence between prey availability and use was assessed by the Chi-square goodness of fit test (χ^2^, log-likelihood ratio *G*). Prey types were ranked in order of relative preference according to their standardized preference index (*B*_*i*_) (the number of used resource units in category *i* in the population divided by the total number of used resource units in the population)—see [52]. To assess positive or negative selection, we constructed Bonferroni’s confidence intervals for individually estimated availability and use of habitat types [52, 53]. For all tests, *α* was set to 0.05, corrected by the number of simultaneous comparisons. The confidence intervals were computed at the 95%, also corrected by the number of simultaneous comparisons.

Prior to any selection analysis, prey species were merged so, that the frequency of occurrence of each newly merged group of species was larger than five, as this is the necessary condition for the χ^2^ test to be valid. This yielded seven groups of prey: (i) large wild ungulates (Himalayan tahr, blue sheep and musk deer), (ii) small wild prey (pika, hare, rat and vole), (iii) mustelids (weasels and marten), (iv) birds, (v) yak, (vi) other large mammals (cow, ox and horse) and (vii) medium-sized domestic animals (goats and dogs). For the size selection analyses, prey species were merged into three groups: (i) "large prey" with a body mass > 40 kg, (ii) "medium prey" with a body mass 10–40 kg and (iii) "small prey" with a body mass < 10 kg [20]. Among prey categories, "wild prey" means blue sheep or Himalayan tahr, musk deer, woolly hare, vole, rat spp., pika, weasel spp. and stone marten, while "domestic prey" includes yak, cow, ox, horse, goat and dog.

## Results

Genetic analyses of the 261 scats revealed that 122 (50%) belonged to snow leopard, 71 (29%) to common leopard, 16 (7%) to grey wolf, 6 (2%) to golden jackal and 16 (7%) to red fox.

### Diet

We analysed the remains of 10 wild and 5 domestic species, including dog, in snow leopard scats from the three study areas (Table 1). A single prey species was recorded in 57% of the scats and 2 and 3 prey species in 37% and 8% of the scats, respectively. Plant material such as grass, leaves and twigs of various scrub species were found in 10% of the analysed scats.

**Table 1:** Relative frequency of different kinds of prey in the diet of snow leopard in the three study areas: Lower Mustang (LM) and Upper Manang (UM) in Annapurna Conservation Area, and Sagarmatha National Park4 (SNP) in both summer and winter: *N*, number of scat samples; RF (%), relative frequency; NA, not available. The most frequently consumed prey at each place and season is marked in bold. Nak is yak calf.

	Lower Mustang, LM		Upper Manang, UM		Sagarmatha National Park, SNP		
	summer	winter	summer	winter	summer	winter	Overall
	N = 17	N = 12	N = 29	N = 19	N = 32	N = 13	N = 122
Species	RF (%)	RF (%)	RF (%)	RF (%)	RF (%)	RF (%)	RF (%)
**Wild animals**	35	73	39	64	62	71	57
Blue sheep	30	**23**	22	**29**	NA	NA	NA
Himalayan tahr	NA	NA	NA	NA	**34**	**41**	**29**
Musk deer	5	0	2	0	9	18	5
Woolly hare	0	9	0	7	2	0	3
Vole	0	9	0	4	2	0	2
Rat spp.	0	14	0	4	2	0	3
Pika	0	5	0	4	2	0	2
Mustelids	0	14	15	15	10	12	11
Birds spp.	0	0		4	2	0	2
**Domestic animals**	65	27	61	32	36	29	43
Yak/Nak	20	14	**37**	14	15	0	19
Dog	0	0	12	0	4	6	4
Cow	10	5	2	0	17	24	9
Ox	0	0		0	0	0	1
Horse	0	0	7	4	0	0	4
Goat	**35**	9	2	14	0	0	6
UI				4	2	0	
Total	100	100	100	100	100	100	100

In the Sagarmatha National Park (SNP), the most frequent species of prey in snow leopard faeces was Himalayan tahr in both winter and summer, followed by cow and musk deer in winter, and cow and yak in summer; weasel spp. and dog were also consistently recorded in both seasons, whilst other small prey occurred only in summer (Table 1).

In the ACA region (in both LM and UM), snow leopard consumed mainly blue sheep, goat and yak in winter, and blue sheep, rat, yak and goat in summer. In both LM and UM, musk deer contributed the smallest percentage to the snow leopard diet and weasel spp. contributed 7–10%. Overall, a higher percentage of wild animals were consumed in winter (69%) and domestic animals in summer (54%), and similar results were obtained at both LM and UM. In SNP, there was a higher percentage of wild prey (62–71%) in the diet of snow leopards in both seasons. Regionally, there were more or less equal percentages of wild *vs*. domestic prey recorded in LM and UM, and the highest percentage of wild prey was recorded in SNP.

In terms of relative biomass consumed, yak was the most important species of prey everywhere in both seasons, contributing about half of the biomass in LM, more than half of the biomass in UM in summer, and being among the most consumed in SNP, together with cow in summer (Table 2). Cow and blue sheep were also important prey in LM (and goat to a lesser extent in summer), cow and Himalayan tahr were important prey in SNP, and horse and blue sheep in UM. In contrast, the contribution of small species of prey to the diet of snow leopard was negligible (below 1% of RBC). In summary, domestic species made up most of the diet of snow leopard, making up 78 ± 4.8% of the biomass consumed and wild prey made up only 22 ± 4.8%.

**Table 2:** Relative biomass of the different kinds of prey consumed (RBC) by snow leopard in three study areas: Lower Mustang (LM) and Upper Manang (UM) in Annapurna Conservation Area, and Sagarmatha National Park (SNP). NA—not available.

		Lower Mustang, LM		Upper Manang, UM		Sagarmatha National Park, SNP		
	Weight	summer	winter	summer	winter	summer	winter	
Species	kg(*X*_*i*_)^a^	RBC (%)	RBC (%)	RBC (%)	RBC (%)	RBC (%)	RBC (%)	Overall
**Wild animals**		19	29	12	22	23	27	21
Blue sheep	50	18	22	11	19	NA	NA	18
Himalayan tahr	50	NA	NA	NA	NA	20	23	
Musk deer	10	1	0	0	0	2	1	1
Woolly hare	4	0	2	0	1	0	1	0
Vole	0.3	0	1	0	0	0	0	0
Rat spp.	0.3	0	1	0	0	0	0	0
Pika	0.3	0	0	0	0	0	0	0
Mustelids	1.5	0	2	1	1	1	1	1
Birds spp.	0.3	0	1	0	0	0	0	0
**Domestic animals**		81	71	88	78	77	73	79
Yak/Napki	200	44	49	67	35	38	29	44
Dog	20	0	0	3	0	1	1	1
Cow	200	22	16	4	0	38	21	21
Ox	200	0	0	0	9	0	4	1
Horse	188	0	0	13	33	0	16	8
Goat	35	15	6	1	2	0	2	3
		100	100	100	100	100	100	100

^a^body weights of the prey were obtained from Lyngdoh et al. (2014)

#### Prey availability

In total, 2,877 independent captures of 15 potential mammalian and 1 avian species of prey were taken over 4,567 camera trap nights. Altogether, the highest *RAI* corresponded to yak, followed by pika, bird, and blue sheep or Himalayan tahr (Table 3). Musk deer were not recorded in LM and UM, and goat in SNP.

**Table 3:** Relative Abundance Index (RAI) of the different kinds of prey determined using camera traps; N = total trap nights and NA—not available.

	Lower Mustang, LM		Upper Mustang, UM		Sagarmatha National Park, SNP		
	winter	summer	winter	summer	winter	summer	Overall
Species	N = 414	N = 1020	N = 321	N = 1000	N = 1255	N = 557	N = 4567
**Wild animals**	**75**	**49**	**56**	**49**	**49**	**79**	**54 **
Blue sheep	13	3	20	15	NA	NA	13
Himalayan tahr	NA	NA	NA	NA	17	18	
Musk deer	0	0	0	0	6	3	1
Woolly hare	3	1	0	0	0	0	0
Vole	1	1	1	0	1	0	1
Rat spp.	1	0	0	0	0	0	0
Pika	30	26	8	11	6	21	16
Mustelids	9	5	8	7	2	5	6
Birds spp.	18	13	20	15	16	30	16
**Domestic animals**	**25**	**51**	**44**	**51**	**51**	**21**	**46 **
Yak/Nak	11	23	35	39	43	14	31
Dog	2	2	1	1	1	0	1
Cow	0	3	0	0	3	3	1
Ox	2	6	4	2	4	4	4
Horse	0	3	1	7	0	0	3
Goat	10	14	3	2	0	0	6
**Total**	**100**	**100**	**100**	**100**	**100**	**100**	**100**

### Prey selection

Selection analysis based on frequencies of occurrences revealed that snow leopard consumed some species of prey disproportionately relative to their availability both during a whole year (χ^2^ = 105.6, df = 6, P < 0.001) and in winter and summer, separately (χ^2^ = 42.9, df = 6, P < 0.001; and χ^2^ = 41.2, df = 6, P < 0.001, respectively). Prey selection pattern changed seasonally (Table 4). Large wild prey were preferred in summer and consumed at random in winter. Mustelids and large domestic cattle were preferred in winter and consumed at random in summer. Small wild prey was avoided in summer and consumed at random in winter. Yak was avoided in winter and consumed at random in summer. Medium sized domestic animals were consumed at random and birds were avoided in both seasons.

**Table 4:** Prey selection by snow leopard in the whole study area, throughout the year and seasonally. Table includes proportions of different kinds of available prey (Πi), Bonferroni’s confidence intervals and standardized preference index (*B*_*i*_) are shown following Manly et al. (2002). The large wild prey category includes blue sheep, Himalayan tahr and musk deer; small wild prey includes pika, hare, rat and voles; Mustelids are weasels and stone marten; large cattle includes cow, ox and horses; medium domestic prey are goat and dogs. In the column “Selection”, (+) means preference, (-) avoidance, and null random choice.

	Available (relative)	Use (relative)	Bonferroni confidence intervals			
Prey category	Πi	oi	Lower	Upper	Selection	*B*_*i*_
**Overall**						
Large wild	0.141	0.344	0.250	0.439	(+)	0.280
Small wild	0.175	0.093	0.035	0.151	(-)	0.061
Mustelids	0.059	0.109	0.047	0.171	null	0.211
Bird spp.	0.164	0.022	0.000	0.051	(-)	0.015
Yak/Nak	0.307	0.191	0.113	0.269	(-)	0.072
Large cattle	0.083	0.137	0.068	0.205	null	0.188
Medium domestic	0.069	0.082	0.043	0.164	null	0.173
Total	1	1				1
**Winter**						
Large wild	0.194	0.333	0.181	0.486	null	0.176
Small wild	0.146	0.188	0.111	0.266	null	0.133
Mustelids	0.061	0.130	0.063	0.197	(+)	0.218
Birds spp.	0.175	0.043	0.003	0.084	(-)	0.025
Yak/Nak	0.322	0.101	0.041	0.161	(-)	0.032
Large cattle	0.050	0.145	0.075	0.215	(+)	0.300
Medium domestic	0.052	0.058	0.012	0.104	null	0.115
Total	1	1				1
**Summer**						
Large wild	0.113	0.351	0.231	0.471	(+)	0.354
Small wild	0.192	0.035	0.000	0.072	(-)	0.021
Mustelids	0.058	0.096	0.038	0.155	null	0.188
Birds spp.	0.157	0.009	0.000	0.027	(-)	0.006
Yak/Nak	0.299	0.246	0.160	0.331	null	0.093
Large cattle	0.102	0.132	0.064	0.199	null	0.147
Medium domestic	0.079	0.132	0.064	0.199	null	0.190
Total	1	1				1.000

At a regional scale, the predatory behaviour of snow leopard was selective at all three study areas (LM: χ^2^ = 24.9, df = 6, P < 0.0001; UM: χ^2^ = 24.9, df = 6, P < 0.0001; and SNP: χ^2^ = 34, df = 6, P < 0.0001), with slight differences in the prey selected (Table 5). The preferred large wild prey (blue sheep and musk deer) in LM, mustelids and medium sized domestic prey in UM, and large wild prey (Himalayan tahr and musk deer), mustelids and large cattle in SNP. In contrast, yak, nak (female yak) and birds were avoided everywhere, and small wild prey were avoided in LM and SNP, but consumed at random in UM.

**Table 5:** Prey selection by snow leopard in the three areas studied. Table includes proportions of the different kinds of available prey (Πi). Bonferroni’s confidence intervals and standardized preference index (*B*_*i*_) are shown following Manly et al. (2002). Prey categories are the same as in Table 4. In the column “Selection”, (+) means preference, (-) avoidance, and null random choice.

	Available (relative)	Consumed (relative)	Bonferroni confidence intervals			
Prey category	Πi	Oi	Lower	Upper	Selection	*Bi*
LM						
Large wild	0.061	0.273	0.092	0.453	(+)	0.472
Small wild	0.296	0.182	0.105	0.259	(-)	0.065
Mustelids	0.059	0.068	0.018	0.118	null	0.122
Bird spp.	0.141	0.045	0.004	0.087	(-)	0.034
Yak/Nak	0.199	0.159	0.086	0.232	null	0.085
Large cattle	0.094	0.068	0.018	0.118	null	0.077
Medium domestic	0.150	0.205	0.124	0.285	null	0.145
Total	1.000	1.000				1
UM						
Large wild	0.167	0.261	0.119	0.403	null	0.159
Small wild	0.107	0.072	0.021	0.124	null	0.069
Mustelids	0.070	0.145	0.075	0.215	(+)	0.210
Bird spp.	0.162	0.014	0.000	0.038	(-)	0.009
Yak/Nak	0.382	0.275	0.187	0.364	(-)	0.074
Large cattle	0.078	0.130	0.063	0.197	null	0.170
Medium domestic	0.034	0.101	0.041	0.161	(+)	0.308
Total	1.000	1.000				1
SNP						
Large wild	0.221	0.471	0.311	0.632	(+)	0.170
Small wild	0.117	0.057	0.011	0.103	(-)	0.039
Mustelids	0.037	0.100	0.040	0.160	(+)	0.213
Bird spp.	0.205	0.014	0.000	0.038	(-)	0.006
Yak/Nak	0.333	0.129	0.062	0.195	(-)	0.031
Large cattle	0.076	0.186	0.108	0.263	(+)	0.193
Medium domestic	0.010	0.043	0.003	0.083	null	0.349
Total	1.000	1.000				1

### Selection by size

In both the year and summer analyses, the sizes of prey consumed did not correspond with that available (overall: χ^2^ = 30.5, df = 2, P < 0.001; summer: χ^2^ = 41.9, df = 2, P < 0.001), but it did in winter (χ^2^ = 0.64, df = 2, P = 0.73). In summer, snow leopard preferred large-sized prey, whilst medium sized prey was consumed according to its availability, though the standardized preference index for medium sized prey was almost double that of large sized and small-sized prey (Table 6).

**Table 6:** Prey size selection by snow leopard in the whole study area. Table includes proportions of different kinds of available prey by size (Πi). Bonferroni’s confidence intervals and standardized preference index (*B*_*i*_) are shown following Manly et al. (2002). Among prey categories, Large includes species weighing above 40 kg (blue sheep, Himalayan tahr, yak/nak, cow, ox, horse), Medium includes prey between 10–40 kg (musk deer, goat and dog) and Small refers to prey weighing below 10 kg (woolly hare, vole, rat spp., pika, weasel spp., stone marten and birds). In the column “Selection”, (+) means preference, (-) avoidance, and null random choice.

	Available (relative)	Consumed	Bonferroni confidence intervals			
Prey category	Πi	Oi	Lower	Upper	Selection	*Bi*
Overall						
Large	0.521	0.617	0.475	0.760	null	0.319
Medium	0.081	0.158	0.051	0.266	null	0.529
Small	0.399	0.224	0.101	0.347	(-)	0.151
Total	1	1				1
summer						
Large	0.509	0.667	0.528	0.805	(+)	0.330
Medium	0.084	0.193	0.077	0.309	null	0.583
Small	0.407	0.140	0.038	0.243	(-)	0.087
Total	1	1				1

Prey choice in terms of size also differed in the three areas (LM: χ^2^ = 7.4, df = 2, P = 0.025; UM: χ^2^ = 11.2, df = 2, P = 0.004; and SNP: χ^2^ = 17, df = 2, P < 0.001). In LM and SNP, large- and medium-sized prey were consumed based on availability and only small prey were avoided (Table 7); in UM, all the size categories of prey were consumed at random.

**Table 7:** Prey selection of snow leopard by size in the three areas studied. Table includes proportions of the different kinds of available prey (Πi). Bonferroni’s confidence intervals and standardized preference index (*B*_*i*_), following Manly et al. (2002). Prey categories are the same as in Table 6. In the column “Selection”, (+) means preference, (-) avoidance, and null random choice.

	Available (relative)	Consumed (relative)	Bonferroni confidence intervals			
Prey category	Πi		Lower	Upper	Selection	*Bi*
LM						
Large	0.352	0.477	0.330	0.624	null	0.392
Medium	0.151	0.227	0.104	0.351	null	0.435
Small	0.496	0.295	0.161	0.430	(-)	0.173
Total	1	1				1
UM						
Large	0.627	0.652	0.512	0.792	null	0.201
Medium	0.034	0.116	0.022	0.210	null	0.667
Small	0.339	0.232	0.108	0.356	null	0.132
Total	1	1				1
SNP						
Large	0.580	0.671	0.533	0.810	null	0.272
Medium	0.060	0.157	0.050	0.264	null	0.615
Small	0.359	0.171	0.061	0.282	(-)	0.112
Total	1	1				1

### Wild *vs*. domestic prey

Excluding birds from the analysis, as they were always avoided, the analysis of the preference for wild *vs*. domestic prey revealed that overall snow leopards preferred wild prey in winter (χ^2^ = 10.3, df = 1, P = 0.001; Table 8), but showed no preference in summer (χ^2^ = 1.43, df = 1, P = 0.23). Regionally (Table 9), snow leopards preferred domestic prey only in SNP (χ^2^ = 7.6, df = 2, P = 0.006). In LM and UM, wild and domestic prey were consumed according to their availability (LM: χ^2^ = 0.66, df = 2, P = 0.417; UM: χ^2^ = 1.6, df = 2, P = 0.212).

**Table 8:** Selection of wild vs. domestic prey by snow leopard in the three areas in winter. Table includes proportions of available prey by size (Πi). Bonferroni’s confidence intervals and standardized preference index (*B*_*i*_), following Manly et al. (2002). Among prey categories, Wild includes blue sheep or Himalayan tahr, musk deer, woolly hare, vole, rat spp., pika, weasel spp. and stone marten and Domestic includes yak, cow, ox, horse, goat and dog. In the column “Selection”, (+) means preference, (-) avoidance, and null random choice.

	Available (relative)	Consumed (relative)	Bonferroni confidence intervals			
Prey category	Πi	Oi	Lower	Upper	Selection	*Bi*
Winter						
Wild	0.487	0.682	0.556	0.807	(+)	0.693
Domestic	0.513	0.318	0.193	0.444	(-)	0.307
Total	1	1				1

**Table 9:** Prey selection by snow leopard in SNP. Table includes proportions of the different kinds of available prey (Πi). Bonferroni’s confidence intervals and standardized preference index (*B*_*i*_), following Manly et al. (2002).

	Available (relative)	Consumed (relative)	Bonferroni confidence intervals			
Prey category	Πi	Oi	Lower	Upper	Selection	*Bi*
SNP						
Wild	0.527	0.362	0.232	0.491	-	0.337
Domestic	0.472	0.638	0.508	0.767	+	0.633
Total	1	1				1

## Discussion

We show that snow leopard consumes a diverse group of prey and its diet varies both regionally and seasonally. We confirmed previous results that snow leopard, like many other large predators, particularly felids, is a generalist, and its choice of prey is not solely determined by prey availability (cheetah: [54]; lion: [55, 56]; tiger: [57–59]; leopard: [29]).

In this study, large wild prey such as tahr and blue sheep were most frequently consumed and preferred. This was consistent in both summer and winter at all of the areas studied. In summer, these were supplemented with medium-sized domestic species such as goats in LM, or yak in UM. This is consistent with what Chetri et al. [28] found in the Central Himalayas in Nepal. In the Tost Mountains (South Gobi, Mongolia), goat and sheep together make up 20% of the snow leopard’s diet [21], and goats alone 40% at Lossar and Rumtse (India) [60]. Our results also confirm the main conclusions of [20] that snow leopard prefers large wild prey weighing 36–76 kg throughout its distribution.

Large domestic animals (yak, cow, ox or horses), were in spite of their local abundance in general, consumed less than expected. They were often preyed upon proportionally to their abundance and sometimes even avoided (but see yak in summer in UM, Table 2). This can be compared with previous studies in Indian trans-Himalayas and Nepal Central Himalayas, where snow leopards prefer horses and goats, avoid yaks and show no preference for sheep or lulu cows [28, 61]. This low frequency of predation of large domestic cattle and yak may be due to several reasons. First, adult yaks may be too large for snow leopards to kill and female yaks protect their calves while grazing, which may make even the calves difficult to hunt. In addition, whilst male adult yaks are unattended throughout a year, breeding female herds with calves are kept at night in summer in temporary corrals close to the sheds of the herders, which may make them less available to snow leopards. Thus, adult yaks and other large animals such as cows, although highly attractive, may be difficult for snow leopards to capture.

Another point is that diets based on the frequency of occurrence of prey tend to overestimate the importance of small prey, which may be consumed more often, but contribute less energy, compared to large prey [62, 63]. Thus, translating our diet data into biomass, yak is the most profitable prey (Table 2) followed by large cattle (mostly cows and horses), whilst the contribution of blue sheep, Himalayan tahr and goats is much lower. Similar results are recorded in Baltistan, Northern Pakistan, where most of the biomass (70%) consumed by snow leopards consists of domestic animals [64].

However, even RBS assessments should be treated with caution, as we do not know whether the size of individual "large cattle" (yak, cows or horses) consumed by snow leopards corresponds to the average size used to calculate biomass eaten. If, as we suggest above in the case of yaks, the largest adults are too difficult to catch and therefore snow leopards mostly consume young animals, then the actual RBC is much lower than that calculated here and in other studies.

The placement of camera traps also needs some discussion. The cameras were placed on ridgelines, near scent-sprayed rocks etc. Some may argue that this is not a random placement, so interpretation of availability requires care since not all species may utilize the same microhabitats or use them in the same way or at the same frequency as snow leopard. We agree that this is possible in theory, but do not believe this has caused a strong bias in the data. We argue that most of the prey species follow the accessible trails or ridgelines (where the cameras were placed) in search for food. Also livestock and domestic dogs can be found here. Thus we do not see any logical reason, why they should be concentrated elsewhere and not where the camera traps were placed, which might have (in theory) caused their underrepresentation in the availability analysis. High frequency of mustelids is interesting and rather surprising, but cannot be explained by camera positioning. The camera sensors were placed 40–50 cm above ground, 2–3 m apart from the anticipated travel path, which should assure capturing both sufficiently large and small mammals. The absence of marmots is not surprising, as they are not found in either of the three study areas.

Some of the results with scats must be taken with caution, as the total number of confirmed scats is rather small (only 12–32 per study area/season). This will, however, not devaluate our main results and only invites further studies in this respect.

Noteworthy is the preference for mustelids in winter and in UM and SNP, and their random predation during summer in LM. This indicates that the hunting behaviour of snow leopard is plastic and it may often also prey on these species, even if their contribution to the overall biomass in the diet is low (Table 2).

Small wild mammals were avoided in summer, or locally in LM and SNP or randomly eaten in winter and in UM, as reported in [60]. Maybe they are not profitable enough as prey. Birds were always avoided, probably because they can fly and therefore are difficult to catch.

Other studies, some of them carried out even in the same area as this study (LM, UM, SNP), often report very different results, such as that e.g. Himalayan marmot is making up 20% of the snow leopard’s diet–this is a species that we did not record. It is reported that small mammals make up 6.3–35% of the snow leopard’s diet, e.g., [13] at UM, [14] at Ladakh, and [27, 65] in SNP; birds are reported making up 14% of the snow leopard’s diet [17] in SNP. All these results are very different from ours. However, these studies most likely overestimated the importance of small prey [66], because none of them used genetic methods for identifying the depositor of the scat and studies solely based on visual identification of samples may be strongly biased. Actually, our molecular analyses revealed that as much as 50% of the scats collected, which looked like snow leopard scats, in fact belonged to other carnivores, which is a number similar to that reported in other studies [21, 23, 26, 39, 67].

Previous results report a high percentage of wild prey in snow leopard’s diet: 73% in Central Nepal [28], 95% at Lingti, India [60], 73% [21] and 78% in Mongolia [68]. In our study, wild prey was significantly more frequent in snow leopard’s diet than domestic prey only in SNP, but not in LM and UM throughout the year, and for the whole study area the difference between percentages of wild and domestic prey was only significant in winter.

However, as the largest prey are commonly domestic animals (yak, cow, ox or horses) livestock surely contribute substantially more in terms of biomass to snow leopard diet. A recent survey carried out in the Trans-Himalayas and Altai, [60] shows that despite the high contribution of livestock to snow leopard diet, wild mammals remain the preferred prey and the critical determinant of snow leopard density. Moreover, their results suggest that, even though snow leopard densities would benefit from an increase in wild ungulates, this would first intensify and later stabilize livestock predation. Interestingly in this context, in our study, livestock was preferred only in SNP throughout the year and in winter in the whole study area (Table 3), precisely where and when wildlife is more frequently available.

Our study provides a comprehensive and reliable dataset on prey selection by snow leopard, rare even for relatively well-studied wide-ranging large carnivores such as tigers [57]. Based on this, we can confirm that both wild large ungulates and livestock are the main prey of snow leopard in the study area, both in terms of percentage of occurrences and biomass. Nevertheless, Himalayan and Central Asian mountains are becoming increasingly livestock-dominated and wild herbivore biomass has been reduced to less than 5% of the livestock biomass in much of Central Asia [68]. Consequently, increasing populations of wild ungulates is as critical a measure for conservation of snow leopard as it is for other carnivore populations [57, 69].

However, such an increase in prey densities is unlikely to slow down livestock predation, which may even increase, especially when livestock is abundant [60]. If so, the local people dependent on livestock may still resort to retaliatory killing of snow leopards [15]. In our experience, this occurs: during 2016, we found one snow leopard in LM with injuries to a hind limb, most likely caused by a trap; the animal died three days later.

In order to prevent retaliation, snow leopard conservation efforts must be accompanied by greater assistance to shepherds and cattle breeders for livestock protection and indemnifying losses caused by predation. In fact, the small financial compensation provided by the government to local people for snow leopard attacks is minute in comparison with the cost of livestock [70]. Therefore, effective snow leopard-human conflict-mitigation programmes, especially oriented towards local herders, should be used to change their altitude in terms of toleration of the presence of snow leopards in their areas. This will improve snow leopard conservation.
